# Promoting Health by Improving Subjective Sleep Quality? Reduction in Depressive Symptoms and Inflammation as Potential Mechanisms and Implications for Trauma-Exposed Persons

**DOI:** 10.3389/fpsyt.2016.00076

**Published:** 2016-04-27

**Authors:** Tamara L. Newton, Rafael Fernandez-Botran

**Affiliations:** ^1^Department of Psychological and Brain Sciences, University of Louisville, Louisville, KY, USA; ^2^Department of Pathology and Laboratory Medicine, University of Louisville, Louisville, KY, USA

**Keywords:** sleep quality, inflammation, traumatic stress, depression, gene expression regulation

Subjective sleep quality refers to perceived ease of falling asleep, staying asleep, and obtaining adequate sleep that leaves one feeling rested. Such perceptions do not necessarily correspond to objective measures of sleep quality provided by polysomnography. Nevertheless, they are the key to diagnosing some sleep disturbances, such as insomnia (1).

Insomnia can be treated, and subjective sleep quality improved, by interventions that help people identify and change beliefs and behaviors that interfere with sleep (2). Livingston et al. (3) examined whether responses to such cognitive–behavioral interventions are associated with changes in the expression of genes involved in the regulation of inflammation.

Participants were 68 active duty United States military personnel (97% males) seeking sleep evaluations. All had been deployed within the previous 18 months and had current diagnoses of insomnia or insomnia comorbid with obstructive sleep apnea. Probable syndromal depression or posttraumatic stress disorder (PTSD) was present in 47 and 28% of participants, respectively.

Participants were assessed before, and immediately after, completing treatment for insomnia (cognitive–behavioral therapy for insomnia) or for comorbid insomnia and obstructive sleep apnea (either sleep education or cognitive–behavioral therapy for insomnia combined with positive airway pressure). Assessments included subjective sleep quality, depression and posttraumatic stress symptoms, and peripheral blood gene expression profiles.

After 3 months of treatment, two clusters of participants were identified, those with any improvement in subjective sleep quality (68% of the sample) and those without any improvement (32% of the sample). The former group, compared to the latter, showed decreases in symptoms of depression from pre- to posttreatment. Microarray data identified 217 genes, whose expression was differentially altered in those participants with improved sleep. Consistent with reduced inflammation, downregulated genes included those for key proinflammatory cytokines [interleukin (IL)-1β, IL-6, and TNF-α] and chemokines (CCL3, 4, and 5). Among the upregulated genes were also inflammation/immune response-associated genes, including several toll-like receptors (TLR1, 4, 7, and 8) and cytokine/chemokine receptors (i.e., IFNγR1 and the IL-8R) as well as stress-related genes. Analysis of the gene expression data using ingenuity pathway analysis (IPA) (4) identified six gene networks, including ubiquitin.

Insomnia is an important clinical problem in its own right. In addition, it is increasingly recognized as a contributory factor in multiple mental and physical health problems (5–7), including those that arise post-trauma (5). Connections between insomnia and health problems may partially reflect the importance of sleep in regulating adaptive and innate immunity (8). Indeed, disturbances in sleep that include insomnia and poor subjective sleep quality are associated with elevations in systemic inflammation (9).

Critically, Livingston and colleagues identified potentially salutary changes in gene expression among trauma-exposed persons who responded favorably to cognitive–behavioral sleep interventions. Their results provoke questions about whether interventions for sleep disturbances might feed-forward to promote better health and protect against the emergence of health problems after trauma exposure. While reduction in the expression of key proinflammatory cytokine/chemokine genes is consistent with reduced inflammation as a potential mechanism, the relevance of changes in the expression of other genes, such as those for immune receptors and stress-related molecules, remains to be further investigated.

Several directions for future research are suggested by this preliminary investigation. Regarding the sample, it will be important to test whether results generalize to sleep disturbances that arise in the context of exposure to other traumatic stressors; sleep disturbances in military personnel are determined by myriad factors that may not apply in other contexts (e.g., time zone changes and potential circadian disruption, long and varying work shifts, and health behaviors used to manage these challenges) (10). Similarly, testing whether these results extend to trauma-exposed women is a high priority, given sex and gender differences in multiple aspects of sleep (11). Regarding the intervention, controlled trials will be necessary to determine whether improved subjective sleep quality is due to the intervention *per se*, as opposed to the passage of time or non-specific factors. A recent randomized controlled trial for later-life insomnia provides evidence for the specific value of cognitive–behavioral interventions, including for salutary changes in gene expression (12). Also, the persistence of the effects over time should be addressed. Regarding the results, because the cognitive–behavioral sleep interventions were associated with improved gene expression profiles in a subset of insomnia patients – those with improved sleep quality after treatment – the next logical step is to identify factors that differentiate treatment responders from non-responders. Also, microarray data of the key genes need to be further validated and more mechanistic studies performed. Finally, the interplay of sleep quality improvements, depression reductions, and gene expression requires attention – what is the order of the causal chain and are reciprocal influences present?

Among trauma-exposed persons, sleep disturbances do not always respond to interventions for PTSD. Even when participants no longer meet diagnostic criteria for syndromal PTSD, clinically significant sleep disturbances may persist (13, 14). Similarly, in observational studies, trauma history helps explain clinically significant sleep disturbances, independent of current mental health symptoms (15). Thus, among trauma-exposed persons, sleep problems may not always be by-products of other mental health problems. The results of Livingston et al. (3) raise the intriguing question of whether specifically addressing such sleep problems, in addition to other post-trauma stress reactions, might yield broader health benefits for trauma-exposed persons.

## Author Contributions

The authors TN and RF-B drafted and revised the manuscript.

## Conflict of Interest Statement

The authors declare that this work was conducted in the absence of any commercial or financial relationships that could be construed as a potential conflict of interest.
